# Variability in the Effectiveness of Two Ornithological Survey Methods between Tropical Forest Ecosystems

**DOI:** 10.1371/journal.pone.0169786

**Published:** 2017-01-10

**Authors:** Thomas Edward Martin, Josh Nightingale, Jack Baddams, Joseph Monkhouse, Aronika Kaban, Hafiyyan Sastranegara, Yeni Mulyani, George Alan Blackburn, Wilf Simcox

**Affiliations:** 1 Operation Wallacea Ltd, Wallace House, Old Bolingbroke, Lincolnshire, United Kingdom; 2 School of Biological Biomedical and Environmental Sciences, Hardy Building, University of Hull, Hull, United Kingdom; 3 Programa de Pós-Graduação em Ecologia, Universidade Federal do Rio Grande do Sul, Av. Bento Gonçalves, Porto Alegre, Brazil; 4 Department of Forest Resources, Conservation, and Ecotourism, Faculty of Forestry, Bogor Agricultural University, Bogor, Jawa Barat, Indonesia; 5 Lancaster Environment Centre, Lancaster University, Lancaster, United Kingdom; University of Delhi, INDIA

## Abstract

Birds are a frequently chosen group for biodiversity monitoring as they are comparatively straightforward and inexpensive to sample and often perform well as ecological indicators. Two commonly used techniques for monitoring tropical forest bird communities are point counts and mist nets. General strengths and weaknesses of these techniques have been well-defined; however little research has examined how their effectiveness is mediated by the ecology of bird communities and their habitats. We examine how the overall performance of these methodologies differs between two widely separated tropical forests–Cusuco National Park (CNP), a Honduran cloud forest, and the lowland forests of Buton Forest Reserves (BFR) located on Buton Island, Indonesia. Consistent survey protocols were employed at both sites, with 77 point count stations and 22 mist netting stations being surveyed in each location. We found the effectiveness of both methods varied considerably between ecosystems. Point counts performed better in BFR than in CNP, detecting a greater percentage of known community richness (60% versus 41%) and generating more accurate species richness estimates. Conversely, mist netting performed better in CNP than in BFR, detecting a much higher percentage of known community richness (31% versus 7%). Indeed, mist netting proved overall to be highly ineffective within BFR. Best Akaike's Information Criterion models indicate differences in the effectiveness of methodologies between study sites relate to bird community composition, which in turn relates to ecological and biogeographical influences unique to each forest ecosystem. Results therefore suggest that, while generalized strengths and weaknesses of both methodologies can be defined, their overall effectiveness is also influenced by local characteristics specific to individual study sites. While this study focusses on ornithological surveys, the concept of local factors influencing effectiveness of field methodologies may also hold true for techniques targeting a wide range of taxonomic groups; this requires further research.

## Introduction

Birds are one of the most widely utilized vertebrate groups for conducting ecological monitoring in tropical forests. This is because they are a well-studied and easily identifiable taxon that are relatively straightforward and cost-effective to survey in the field [1,2]. Additionally, birds can also act as high-performance ecological indicators of other, more cryptic, taxonomic groups [3,4]. Numerous methodological approaches for surveying birds in tropical forests have been developed in recent decades, but the two most commonly used techniques are point count and mist net surveys [1,5]. Point counting involves completing timed counts across a series of survey stations, during which all species detected (usually within a fixed radius) are recorded [1]. Mist netting involves using fine mesh nets to monitor bird communities by determining local richness and also abundance via mark-release-recapture analysis [6]. Both methods have been frequently used to systematically examine richness of bird communities within and between tropical forest ecosystems [7–9].

Numerous detailed comparisons of the relative effectiveness of these methods have been completed in tropical ecosystems, especially within the neotropics [10–13]. This has allowed generalized conclusions regarding the relative strengths and weaknesses of these techniques to be drawn, at least in the context of individual study sites. For example, point counts are known to be influenced by external environmental factors and observer skill, and considered less effective at detecting furtive species or species with peak activity times that differ from the timings of the point counts. Mist nets, meanwhile, are usually considered less efficient than point counts and are similarly limited by external environmental factors, as well as variations in bird behaviour and the limited capture range of the nets (typically from ground-level to about 3 m above the forest floor) [14,15]. Understanding these limitations has allowed for broad recommendations regarding how to employ these methodologies appropriately.

All these previous studies, however, have invariably been conducted within a single forest ecosystem, almost exclusively within the neotropics, and as such their results are only directly relevant to that specific study site. This is a matter of concern, as structural composition of tropical forests varies considerably on a global scale [16], with consequential disparities in the structures of bird communities inhabiting these forests [17]. It is therefore possible that the well-defined strengths and weaknesses of point count and mist net methodologies may be more pronounced in some tropical forest ecosystems than others, depending on local habitat and avian community structure. This could mean these survey methods are more appropriate for use in some ecosystems than others; however this potential source of variability remains unexplored.

In this study we aim to examine how the effectiveness of point counts and mist nets can be environmentally mediated by applying consistent methodologies to two widely separated and biogeographically dissimilar tropical forest ecosystems; a Mesoamerican cloud forest and an insular lowland forest in Indonesia. This will allow for the first critical evaluation of ecological influence on two important methodologies between widely separated geographical sites.

## Materials and Methods

### Ethics statement

All necessary permits and approvals for this study were obtained from the National Parks service in Honduras and RISTEK in Indonesia. All mist netting was undertaken by licensed bird banders holding UK A or C class licenses.

### Study sites

**Fig 1:**
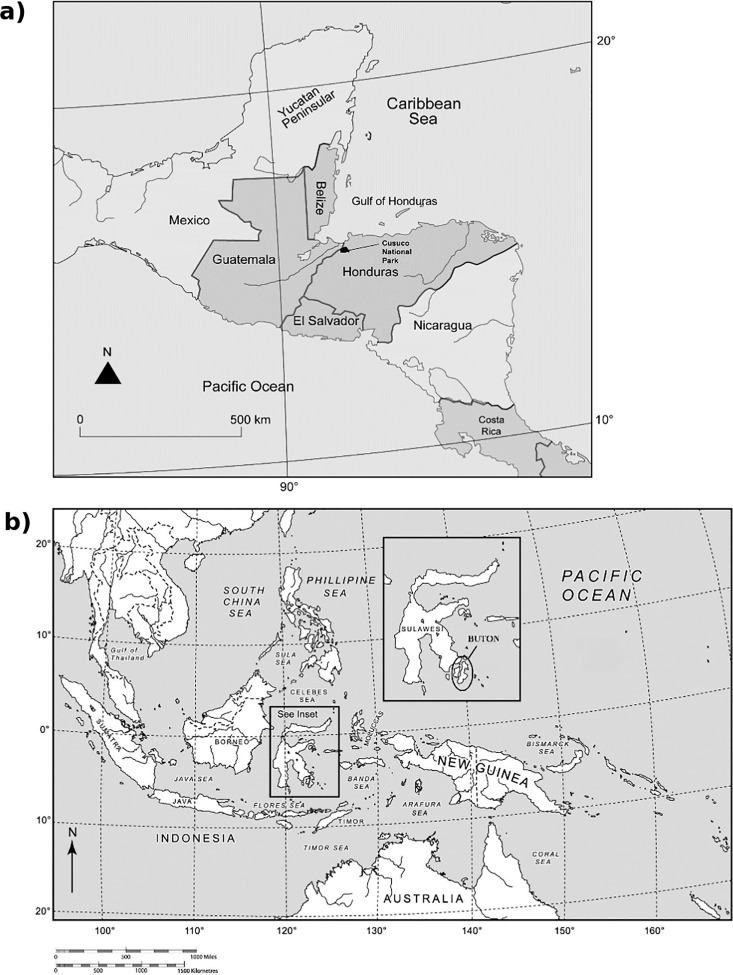
**Maps showing the localities of (a) Cusuco National Park within Mesoamerica, and (b) Buton Island within South-East Asia**.

Fieldwork was conducted in two widely separated tropical forest ecosystems within the biodiversity ‘hotspots’ [18] of Mesoamerica and Wallacea. The Mesoamerican field site was located in Cusuco National Park (CNP), a 23,440 ha cloud forest reserve located in north-western Honduras (Fig 1A). CNP is located within the Sierra de Omoa, encompassing an altitudinal range of 500–2242 m above sea level [19]. Various vegetation classifications occur across the altitudinal gradient of CNP, including lowland forest at lower altitudes (500–1200 m), pine-oak forest and tropical montane cloud forest in the middle-upper altitudinal zones (1200–2000 m), and ‘Bosque enano’ elfin forest at altitudes >2000 m [19]. Annual precipitation in CNP is 2788 mm, with mean monthly temperatures ranging from 12.9°C in December to 20.2°C in April [12,20].

The Wallacean forest site was located in Buton Forest Reserves (BFR), a 189,000 ha area of lowland tropical forest located on Buton Island, Sulawesi, Indonesia. (Fig 1B.). Two reserves occur in the area; Lambusango Forest Reserve in the central-southern part of the island, and Buton Utara Nature Reserve in the north. Altitude varies from between 0–200 m in coastal areas to around 400–800 m in the reserve’s interior [21]. Vegetation is broadly classified as seasonal tropical lowland forest, although various successional stages of disturbance occur, including near-primary forest, well-regenerated secondary forest, and disturbed secondary forest [12]. Mean annual rainfall on Buton ranges between 1,500 and 2,000 mm, peaking between April and June. Mean monthly temperatures are fairly consistent, ranging between 25°C and 27°C [22].

### Data collection

A comparison of point count and mist netting methodologies, broadly based on those employed in a different study [13] was previously completed in CNP between June–August 2007 [12]. Data from this study was used again here, with the same survey protocols being employed in BFR between June–August 2014 to allow for meaningful comparisons of the two techniques between ecosystems.

Point count stations at both sites were located along linear transects, with each station being spaced 300 m apart. Transects contained between four and seven stations at both sites. Each morning ornithologists would walk a transect between dawn and a maximum of three hours after dawn, stopping at each station to conduct 10-minute circular plot point counts [1]. During these counts all species seen and heard within a 50 m radius were recorded, excluding species flying above the canopy. A total of 126 stations were surveyed in CNP, and a total of 77 stations were surveyed in BFR (though the number of stations included in this analysis was subsequently standardized—see field data analysis below). Each station was surveyed three times in their respective field seasons. Point counts were conducted by experienced ornithologists trained in local bird vocalizations. TM led point count surveys in both CNP and BFR.

Mist netting was conducted at the same localities as the point count stations. Each morning three 2.6 m x 20 m 36 mm mesh nets were set up in the understorey of either one or two stations. A mesh size of 36 mm was used as this size is capable of capturing a wide range of species occurring in most ecosystems [23]. Mist nets were opened half an hour after dawn each morning and checked every 20 minutes for a four-hour period, with all species caught being identified, banded, and released. Mist netting was completed at a total of 26 stations in CNP, and 22 stations in BFR, with sampling at each station being repeated once. The number of stations included in this analysis was subsequently standardized (see field data analysis below). Mist netting in both CNP and BFR was led by WS.

### Checklist construction and community structure analysis

As well as compiling data from the two survey protocols, overall checklists of all species known to occur in CNP and BFR were constructed using all available ornithological records from the two sites, sourced through formal survey work and opportunistic sightings. Sum total effort in both sites was sourced from eight weeks of survey work conducted annually over a ten-year period commencing in 2005. These species checklists largely followed those elsewhere for CNP [24], and BFR [22,25], although we only included resident species or migrants that were present during the June-July study period. We also excluded a total of 33 species from this CNP checklist (representing records considered by long-term Operation Wallacea ornithology staff here to be of uncertain and/or dubious origin), and included four new records made since its publication.

Once checklists from both sites were completed, we analyzed the species assemblages of each to allow for comparisons in bird community structure. We first determined the number of avian families present at each site, and compared the number of species within each family using a Mann-Whitney U test [26]. We then subdivided avian communities in CNP and BFR into body-size, feeding guild, and foraging height strata categories, largely following categories defined in a previous study [13]. Data for subgroup classifications were sourced from regional guides for CNP [27] and BFR [28], as well as individual species entries on an authoritative online database [29]. For body-size, we classified species with an average body length of >30 cm as ‘large’, 20 cm– 30 cm as ‘medium’, and <20 cm as ‘small’, following previous precedence for these categorizations [30]. Body length was used as a gauge of size due to the lack of available body mass data for many Wallacean species [30]. Species were categorized by feeding guild according to whether they were predominately classed as carnivores/carrion eaters, insectivores, frugivores / granivores, or nectarivores. For foraging height strata, species were divided accordingly to whether they predominately forage food in the air, canopy, mid-storey, understorey or on the ground/water. We calculated the proportions each of these subgroups comprised of total bird communities in each study site, and compared these proportions with a series of χ^2^ tests.

### Field data analysis

Due to the unequal sampling effort between CNP and BFR, we first removed all data from 49 randomly selected point count stations and four mist netting stations in CNP using a random number generator in Microsoft Excel 2013. This gave an equal-sized sample of 77 point count stations and 22 mist net stations in both sites. To ensure the subsets of data from CNP we analysed were typical exemplars of the possible subsets of these data, we used randomized bootstrapping techniques [26] to plot the distribution of proportions of species detected by both methods over 1000 replicates of randomly removing surveys from the parent dataset, and compared this against the percentage of species detected by both methods in our subsamples (see S1 File for full methods).

Once these representation tests were complete, we calculated the percentage of unidentified individuals in the datasets produced by both methods at both sites. These unidentified records were excluded from further analysis. We then compared the number of species and number of individuals caught per station at each site (after all replicates were complete) using Mann-Whitney U tests, considering each capture method separately. We also calculated the percentage of total known bird species detected by both methods, and compared ratios of detection for each method in each site using χ^2^ tests, and compared detection ratios for all avian subgroups described between sites for each method using a further series of χ^2^ tests with the Yates correction for continuity [26].

To examine which factors consistently affected species' detection probabilities, we tested a series of occupancy models. In traditional occupancy models, two parameters are estimated from survey data: ψ, the probability of occurrence, and *p*, the probability of detection [31]. In our study, we assumed that our checklists represent complete inventories of each site's avifauna, and therefore that each included species' ψ = 1. We then used logistic regression to predict *p* (the probability of detecting each species). Mist net and point count surveys were considered separate Bernoulli trials, as they were never conducted simultaneously. Therefore, for each species our data contained a 0 or 1 reflecting detection by point counts, and another reflecting detection by mist netting. To find the model with the best combination of explanatory power and parsimony, we used Akaike's Information Criterion (AIC) [32]. The global model contained all explanatory variables, the study site, survey method, an interaction between site and method, and species' diet, size and foraging height, excluding any that were non-significant. We also fitted all sub-models containing site, method and their interaction; these variables being central to our research questions. Additionally, we tested models without the interaction, with just one of site or method independently, and finally a null (intercept-only) model. Any model containing non-significant terms was excluded from the analysis. Models were constructed using R version 3.2.2 [33].

Next, we examined how accurately results derived from both methods in both sites could be used to calculate estimators of total species richness in CNP and BFR. This was done by generating ACE, Chao 2 and MMMeans nonparametic species estimators using 100 randomization runs in EstimateS Version 9.1.0 [34], these being considered appropriate estimators for bird communities in tropical forests [35]. We calculated a mean value from these three estimators as a ‘true’ species richness estimate, given that the effectiveness of different estimators varies depending on dataset composition [9].

Finally, we assessed the efficiency of each method by constructing species effort curves plotting the number of person-hours invested against the percentage of total species detected by each method in each site. Point count efficiency curves were calculated on the basis of a single observer working alone. Mist net efficiency curves were calculated on the basis of two observers working together, and included time taken to set up and take down nets.

## Results

**Fig 2:**
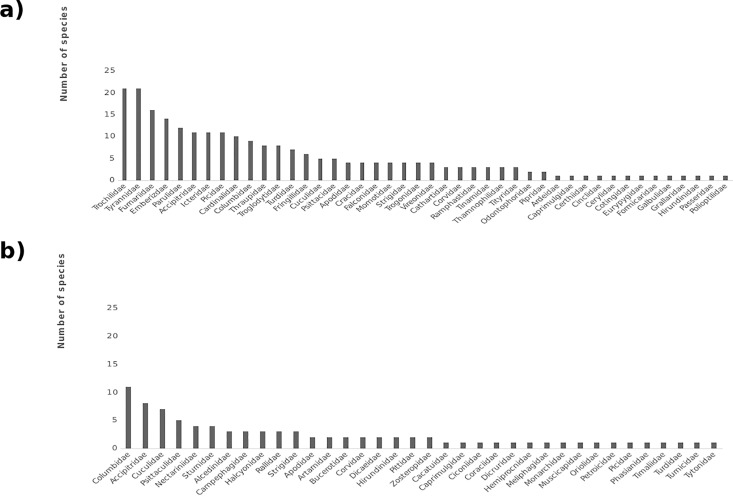
**Graphs showing the number of species in each bird family on the inventory checklists for a) Cusuco National Park (CNP), Honduras, and b) Buton Forest Reserves (BFR), Indonesia**.

Overall checklists totaled 238 species for CNP and 87 species for BFR. These species were split between 44 families in CNP and 36 families in BFR (Fig 2). Families in CNP were significantly more speciose on average than families in BFR (U = 492.5, p <0.01). Table 1 displays the proportions different avian subgroups comprise of overall bird communities in the two sites. CNP is shown to possess significantly higher proportions of small-bodied species, understorey foragers and insectivores (all χ^2^ p-values <0.05), while BFR possesses significantly higher proportions of large-bodied species, aerial and canopy foragers and carnivores (all χ^2^ p-values <0.05).

**Table 1:** Percentage contributions of body size, height strata and dietary subgroups to total community composition in Cusuco National Park (CNP), Honduras, and Buton Forest Reserves (BFR), Indonesia.

		CNP	BFR
**Body size**	**Large**	(44) 18.49%	**(33) 37.93%
	**Medium**	(60) 25.21%	(26) 29.89%
	**Small**	**(134) 56.30%	(28) 32.18%
**Height strata**	**Aerial**	(5) 2.1%	*(7) 8.05%
	**Canopy**	(49) 20.59%	**(36) 41.38%
	**Mid-storey**	(90) 37.82%	(26) 29.89%
	**Understorey**	**(79) 33.19%	(10) 11.49%
	**Ground**	(15) 6.3%	(8) 9.1%
**Diet**	**Carnivores/Carrion**	(22) 9.24%	*(19) 21.84%
	**Insectivores**	**(135) 56.72%	(35) 40.23%
	**Frugivores/Granivores**	(58) 24.37%	(28) 32.18%
	**Nectarivores**	(23) 9.67%	(5) 5.75%

Bracketed numbers indicate the number of species representing each percentage.

Values in bold indicate a significantly higher proportion in the corresponding study site compared to the other study site.

Values indicated * had a χ^2^ test p-value <0.05.

Values indicated ** had a χ^2^ test p-value of <0.01.

Bootstrap analyses show that species detection success in our subsample of data from CNP was statistically representative of typical subsets of the overall dataset (S1 File).

A total of 1881 individual birds were detected by survey work in our subsample of CNP data, with 1454 individuals from 97 different species being detected by point count and 427 individuals comprised of 73 species being detected by mist nets. A total of 2064 individual birds were detected by survey work in BFR, with 2037 individuals from 52 species being detected by point counts and 27 individuals comprised of six species detected by mist nets. These totals do not include unidentified contacts (birds which were registered by surveyors, but could not be confidently identified to a species level). A total of 9.2% and 1.2% of point count contacts were classed as unidentified in CNP and BFR respectively, while 100% of all mist net captures were identified at both sites. A summary of all survey data is provided in S2 File.

Point counts detected a mean of 18.89 ± 6.2 individuals per station in CNP compared to 26.62 ± 15.96 individuals in BFR (U = 2070, p ≤ 0.01), and a mean of 10.17 ± 3.21 species per station in CNP compared to 10.22 ± 4.70 species in BFR (U = 2933, p > 0.05). Mist nets detected a mean of 19.41 ± 8.16 individuals per station in CNP compared to 1.23 ± 1.57 individuals in BFR (U = 3.01, p ≤ 0.01), and 9.55 ± 6.34 species per station in CNP compared to 0.91 ± 0.81 species in BFR (U = 8, p ≤ 0.01).

Point counts detected a total of 40.76% of all species known to occur in CNP, and 59.77% of all species known to occur in BFR (Table 2). Mist nets detected 30.67% of all species known to occur in CNP and 6.9% of all species known to occur in BFR (Table 2). Point counts detected a significantly higher proportion of species overall in BFR compared to CNP (χ^2^ = 8.5, p <0.003), while mist nets detected a significantly higher proportion of species overall in CNP than BFR (χ^2^ = 18.3, p <0.001).

**Table 2:** Percentages of total bird communities and body size, height strata and dietary community subgroups detected by point counts and mist nets in Cusuco National Park (CNP), Honduras, and Buton Forest Reserves (BFR), Indonesia.

		Point counts		Mist nets	
		CNP	BFR	CNP	BFR
**Total community**		(97) 40.76%	**(52) 59.87%	**(73) 30.67%	(6) 6.9%
**Body size**	**Large**	(22) 50%	(20) 60.61%	(1) 2.27%	(0) 0%
	**Medium**	(27) 45%	(15) 57.69%	(7) 11.67%	(2) 7.69%
	**Small**	(48) 35.82%	*(17) 60.71%	**(65) 48.51%	(4) 14.29%
**Height strata**	**Aerial**	(1) 20%	(4) 57.14%	(0) 0%	(0) 0%
	**Canopy**	(18) 36.7%	**(25) 69.44%	(3) 6.12%	(0) 0%
	**Mid-storey**	(37) 41.11%	*(17) 65.38%	*(23) 25.56%	(1) 3.84%
	**Understorey**	(35) 44.30%	(4) 40%	(46) 58.23%	(4) 57.14%
	**Ground**	(6) 40%	(2) 22%	(1) 6.67%	(1) 9.1%
**Diet**	**Carnivores/Carrion**	(5) 22.72%	(6) 31.58%	(0) 0%	(2) 10.53%
	**Insectivores**	(60) 44.44%	(20) 57.14%	*(39) 28.9%	(3) 8.57%
	**Frugivores/Granivores**	(24) 41.38%	**(23) 82.14%	(13) 22.41%	(1) 3.57%
	**Nectarivores**	(8) 34.78%	(3) 60%	**(21) 91.3%	(0) 0%

Bracketed numbers indicate the number of species representing each percentage.

Values in bold had a significantly higher proportion of species detected in the corresponding study site compared to the other study site.

Values indicated * had a χ^2^ test p-value of <0.05.

Values indicated ** had a χ^2^ test p-value <0.01.

On the community sub-group level, point counts detected significantly higher proportions of small-bodied species, canopy and mid-storey species, and frugivores / granivores in BFR compared to CNP (all χ^2^ p-values <0.05), while mist nets detected significantly higher proportions of small-bodied species, mid-storey species, and insectivores and nectarivores in CNP compared to BFR (all χ^2^ p-values <0.05) (Table 2). Point counts detected a higher overall proportion of species in all subgroups in BFR compared to CNP (with the exception of understorey and ground-foraging species), while mist nets detected a higher overall proportion of species in all subgroups in CNP compared to BFR (with the exception of carnivores and ground-foraging species) (Table 2).

The best occupancy model for predicting species detection was the global model, which contained site, method, and interaction between those two terms, as well as diet and foraging height (Table 3). It showed that birds were more likely to be detected in BFR than in CNP, but were less likely to be detected using mist nets than point counts. The interaction term revealed that mist nets performed more poorly in BFR than in CNP (Table 4). Independent of survey method or location, there was a consistent bias in detection towards understorey birds, and away from carnivorous species. All models considering species' ecology were superior to the model containing only method (Table 3). Body size was not significant in the global model, and site was not significant without an interaction with method, so models with these terms were excluded from the model-selection procedure.

**Table 3:** AIC ranking of candidate logistic regression models for predicting detection rates for of bird species by point counts and mist nets in Cusuco National Park (CNP), Honduras, and Buton Forest Reserves (BFR), Indonesia.

Model	AIC	ΔAIC
Site + Method + Site*Method + Height + Diet	747.8	-
Site + Method + Site*Method + Height	757.2	9.4
Site + Method + Site*Method + Diet	762.4	14.6
Site + Method + Site*Method	784.1	36.3
Method	812.8	65.0
null	844.3	96.5

**Table 4:** Parameter estimates from the top-ranked logistic regression model for predicting detection of bird species by point counts and mist nets in Cusuco National Park (CNP), Honduras, and Buton Forest Reserves (BFR), Indonesia. For each categorical predictor variable the number and percentage of species in each category is shown, with the odds ratio of detection for a species in that category, the 95% confidence intervals of that odds ratio and the results of a z-test of the significance of the effect of that category.

Variable	Level	N	%	OR	2.5% CI	97.5% CI	Z	P
**Intercept**	-	-	-	0.63	0.44	0.91	**-2.45**	**0.014**
**Site**	CNP	238	73.2%	1.00	-	-	-	-
	BFR	87	26.8%	3.80	2.17	6.82	**4.58**	**<0.001**
**Method**	Point count	149	45.8%	1.00	-	-	-	-
	Mist net	79	24.3%	0.62	0.41	0.92	**-2.39**	**0.017**
Site * Method	BFR * Mist net			0.07	0.02	0.18	**-5.14**	**<0.001**
**Height**	Aerial	12	3.7%	0.36	0.10	1.07	-1.74	0.08
	Canopy	85	26.2%	0.82	0.48	1.37	-0.76	0.44
	Ground	26	8.0%	0.47	0.20	0.99	-1.91	0.06
	Mid-storey	116	35.7%	1.00	-	-	-	-
	Understorey	86	26.5%	2.10	1.32	3.38	**3.09**	**0.002**
**Diet**	Carnivore	41	12.6%	0.29	0.13	0.58	**-3.33**	**<0.001**
	Frugivore	67	20.6%	1.03	0.62	1.70	0.11	0.91
	Granivore	19	5.8%	0.71	0.33	1.49	-0.87	0.38
	Insectivore	170	52.3%	1.00	-	-	-	-
	Nectarivore	28	8.6%	1.48	0.77	2.88	1.18	0.24

Table 5 shows that the mean estimate of non-parametric richness estimators based on point count sampling effort in CNP predicts 123 species to be present (51.7% of known richness), while the mean estimate from BFR predicts 55 species to occur (63.2% of known richness). Table 6 shows that the mean estimate of non-parametric richness estimators based on mist net sampling effort in CNP predicts 94 species to be present (39.5% of known richness), while the mean estimate in BFR predicts 7 species to occur (8.05% of known richness).

**Table 5:** Comparisons of non-parametric species richness estimators in Cusuco National Park (CNP), Honduras, and Buton Forest Reserves (BFR), Indonesia, for point counts. ACE, CHAO2, and MMMeans are non-parametric species estimators [32].

	CNP	BFR
Sample size	77	77
Species observed	97	52
Individuals observed	1454	2064
ACE	136.45	55.54
Chao2	131.55	57.57
MMMeans	101.92	52.66
Average of species richness estimates	**123**	**55**

**Table 6:** Comparisons of non-parametric species richness estimators in Cusuco National Park (CNP), Honduras, and Buton Forest Reserves (BFR), Indonesia, for mist nets. ACE, CHAO2, and MMMeans are non-parametric species estimators [32].

	CNP	BFR
Sample size	22	22
Species observed	73	6
Individuals observed	427	27
ACE	89.68	7.34
Chao2	88.75	6.48
MMMeans	103.8	8.57
Average of species richness estimates	**94**	**7**

**Fig 3:**
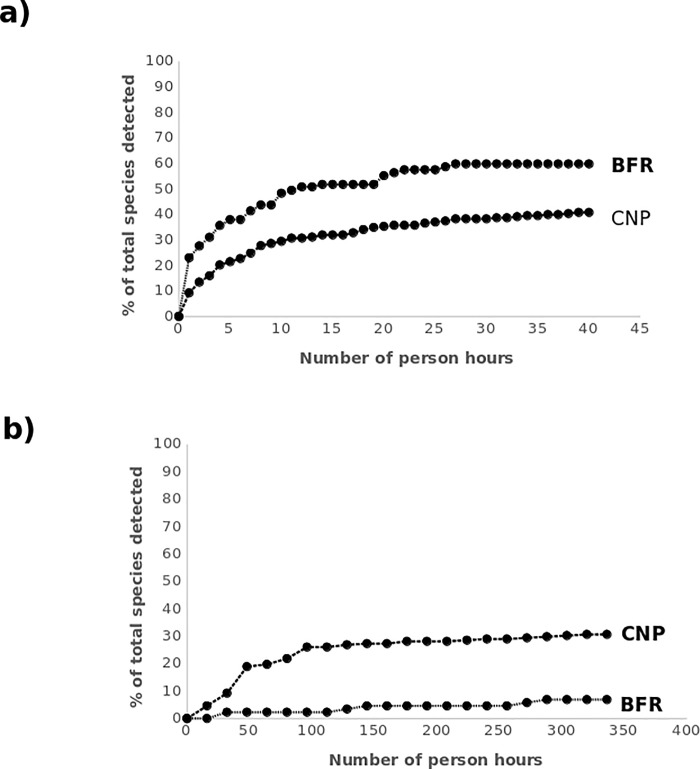
**Species efficiency curves plotting number of person hours invested against percentage of bird communities detected for a) point count surveys, and b) mist net surveys in Cusuco National Park (CNP), Honduras, and Buton Forest Reserves (BFR), Indonesia**.

Species efficiency curves in Fig 3A. indicate that a greater proportion of total species present are detected with increasing person-hours investment by point counts in BFR compared to CNP, with both curves becoming relatively stable after 30 hours of surveying. Fig 3B. indicates that mist nets in CNP detect a far greater proportion of species with increasing person-hours investment compared to BFR.

## Discussion

Our results indicate that the effectiveness of our survey methods differs significantly between study sites. Point counts in BFR detected a higher proportion of overall community richness than point counts in CNP, produced more accurate species richness estimators, and had a much lower proportion of unidentified contacts. Mist nets in CNP, conversely, far outperformed mist nets in BFR. They detected significantly more individuals and species per station and a far greater proportion of the overall bird community. Mist netting in BFR proved generally to be a highly ineffective methodology, detecting an average of less than one species per station, producing entirely unrepresentative species richness estimates, and representing a very inefficient investment of survey effort.

Our results show regional differences occur between both methods' effectiveness in detecting various functional subgroups, as well as regional differences in the relative frequency of these subgroups within the bird community. However, these detection differences were still apparent in models controlling for species' subgroup membership, suggesting that variations in methodological effectiveness are also related to other differences in habitat and bird community structure.

As a cloud forest ecosystem, CNP is characterized by a relatively low canopy, with 80% of sites surveyed having a canopy level of <15 m [12] and a dense, humid understorey which possesses an unusually high proportion of total ecosystem biomass compared to most other tropical forests [36]. This is a product of the steep terrain where these forests occur and the presence of enveloping cloud banks as a major source of precipitation [37]. The high proportions of biomass and feeding resources within the lower levels of cloud forests may facilitate a fairly ‘bottom-heavy’ bird community structure, with CNP possessing a significantly higher percentage of understorey species compared to BFR; a sub-group that have previously been found to possess a relatively low detection rate by point counts [10,15]. Additionally, a large proportion of understorey species in CNP (26.6%) are hummingbirds (Trochilidae), a group considered challenging for point count methodologies due to their weak calls, small size, and swift movement [10]. The high hummingbird richness in CNP could therefore perhaps contribute towards both the relatively high number of unidentified contacts and the low detection rates of nectarivores here. Mist netting methodologies, conversely, may benefit from the habitat and bird community structures found in cloud forest ecosystems. The low canopy of these forests combined with the high proportion of understorey species found here may mean that a larger ratio of species occur within a mist nets capture zone than is typical of other tropical forests. Over 50% of species found in CNP are also small-bodied; this is relevant as small body size has been positively correlated with mist net capture rate in previous studies [13].

While we hypothesize CNP as representing a ‘bottom-heavy’ bird community, similar arguments could class BFR as a 'top-heavy’ bird community. The forest canopy here is comparatively tall, averaging 30 m [38] compared to < 15 m in CNP. A total of 41.4% of species found in BFR are predominantly found in these tall canopies (significantly higher than the proportion of canopy species in CNP), with only 20.7% of species primarily foraging in the understorey or on the ground; approximately half the proportion found in CNP. A key reason for this ‘top-heavy’ ecosystem in likelihood relates to the concentration of feeding resources found in the canopy here. Fruiting figs represent the most important single feeding resource in Wallacean forests [39]. Their canopy-level crops form a crucial part of the diet of not only the 32% of the total bird community represented by frugivores (78.6% of which are predominantly canopy species), but also insectivores who eat invertebrates attracted to the figs [39]. Although detailed botanical information on BFR and other Wallacean forests remains scant, personal observations from all authors suggest most other fruit and nectar food resources occur at the mid-storey or canopy level. Very few flowering plants were observed in the understorey here—a circumstantial explanation for why only around one-fifth of species in BFR forage in the lower habitat strata.

The high proportion of canopy foraging species in BFR could perhaps be predicted to have a negative effect on point count effectiveness, as it has been hypothesized that species become harder to detect with counts in the upper strata of tropical forests [10]. However, in BFR a higher proportion of canopy birds (69.4%) were detected than for any other habitat strata. This may relate to only a few canopy-level birds in BFR being small-bodied (11.1%), the remainder being large or medium-sized birds, most of which possess distinctive, highly audible calls [28]. Indeed, most species in BFR were considered by observers to have fairly distinctive calls generally. This might relate to the less speciose bird families found here (Fig 2). The authors’ experience suggests that species within highly diverse families can often be more difficult to identify by counts than species in low diversity families, as these can entail having to distinguish between many species which are very similar in call and appearance. This is especially true regarding families comprised of small-bodied species with soft or otherwise hard to distinguish calls; CNP, for example, supports 21 Trochilidae species and 16 Furnariidae species, and eight families here are represented by >10 species. BFR, on the other hand, supports only 1 family comprised of >10 species (Columbidae), which is largely comprised of large-bodied species with distinctive calls, and 83.3% of families here are represented by three species or less. It could therefore be argued that the soundscape environment of BFR is considerably less complex than that found in CNP, and thus might account for the lower rate of unidentified contacts and the higher proportion of species detected overall.

While the ‘top-heavy’ bird community of BFR does not seem to be significantly detrimental to point count methodologies, it is almost certainly a major contributor to the ineffectiveness of mist net methodologies here. Given that around 80% of the bird community in BFR are mid-storey or canopy species, the very few captures achieved by the nets here is likely due to most species simply being beyond the capture range of the nets, the ineffectiveness of mist nets for capturing birds inhabiting upper habitat strata having been well documented [10,13,14]. The high proportion of large-bodied species in BFR may also contribute to the ineffectiveness of nets here–larger species having been found to be less susceptible to mist net captures [10,15].

## Conclusion

In summary, this study demonstrates that the effectiveness of bird survey methodologies is strongly influenced by habitat structure and community composition, resulting in significant variation between tropical ecosystems. Results from CNP and BFR act as effective examples for this hypothesis, although similar findings might be apparent elsewhere. At least 119 distinct tropical forest ecoregions have been identified [40], each possessing different structural characteristics and supporting different bird community compositions which may influence methodological effectiveness.

While the generalized strengths and weaknesses of point count and mist netting methodologies described in previous studies should be considered broadly accurate, our results indicate that assuming these strengths and weaknesses have equal implications within any tropical forest could lead to ineffective survey strategies. Methodological textbooks often provide broad assumptions that the employment of point count or mist net survey protocols will in likelihood detect an approximate percentage of species present in a given area [1,41], and researchers often thus employ both methods extensively with the assumption that this approach will harness the general strengths of each to detect the maximum number of species present in a given area [7,9]. However, our results suggest that by first examining the specific characteristics of their focal study site, prospective project planners may be able to design more efficient survey protocols. For example, in ‘bottom heavy’ ecosystems with low canopies, dense understories, and high proportions of small-bodied, cryptic species (such as that found in most cloud forests), researchers may benefit from prioritizing mist netting methodologies as a primary method and use point counting as a subsidiary method. Conversely, in ‘top-heavy’ ecosystems with uncomplicated soundscapes (such as those found on many South-east Asian islands), researchers may achieve better results by focusing on point count surveys and investing less time in mist netting (or even avoiding the methodology entirely if large-bodied canopy feeders are as predominant as they are in BFR).

Finally, it is worth highlighting that, while this study focusses on bird surveys, the same considerations of methodological effectiveness being influenced by local community compositions and habitat structures in likelihood apply to surveyors targeting other taxonomical groups. This is an avenue of enquiry that would benefit from further research.

## Supporting Information

S1 File S1_File.doc. Summary of tests determining the influence of random removal of survey points. (DOCX)

S2 File S2_File.csv. Summary of field survey data from Buton Forest Reserves and Cusuco National Park. (CSV)
