# Supplementary material for: Variability in the Effectiveness of Two Ornithological Survey Methods between Tropical Forest Ecosystems
Source: PLoS One. 2017 Jan 10;12(1):e0169786. doi: 10.1371/journal.pone.0169786 (PMC5224979; doi:10.1371/journal.pone.0169786)
Supplement: S1 File — (DOCX) [file pone.0169786.s001.docx]

**Supporting appendix SS1: Summary of tests determining the influence of random removal of survey points.**

The number of species detected by any sampling method is necessarily dependent on survey effort. For this reason, the number of samples included at each site was standardised by randomly removing surveys from whichever location had the greater number (CNP in both cases). However, different random subsamples may vary amongst themselves in terms of parameters of interest, in this case the number of species detected.

For this reason, we confirmed that the random subsample analysed in detail in our paper was a typical representation of the distribution of possible subsamples that could have been drawn from these data. Drawing 1000 random subsamples from the CNP data (without replacement, as there were sufficient unique combinations available), we compared the distribution of these subsamples with the ones examined in the main analyses of the paper, to confirm that our conclusions were not unduly influenced by this process.


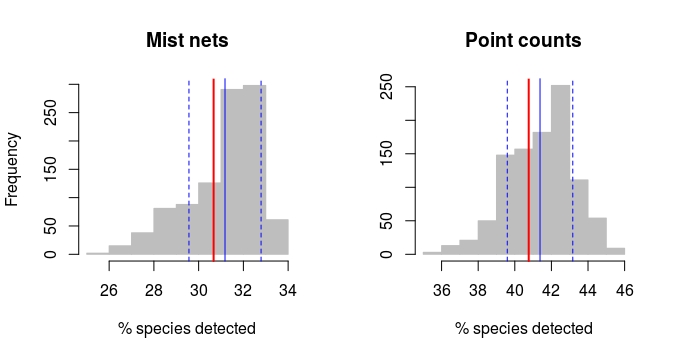
**Figure S1**: Distributions of 1000 randomly-drawn subsamples of surveys from Cusuco National Park, Honduras. The red vertical line indicates the percentage of species detected in the subsample analysed in the paper. The blue vertical line is the mean percentage of species detected in each of the 1000 random subsamples; the dotted blue lines depict an interval of 1 standard deviation either side of this mean.

For both mist nets and point counts, the subsample analysed in the paper detected a slightly lower percentage of species that the mean of all random subsamples, but well within the range of 1 standard deviation from the mean.

The analysed subsample of mist net surveys detected 30.7% of all species, compared with a mean (± sd) of 31.2% (± 1.6) for all random subsamples. The analysed subsample is therefore within half a standard deviation of the mean for all subsamples. By contrast, mist nets in BFR detected 6.9% of species – well below the range of any random subsamples of the CNP surveys, demonstrating that our conclusions would hold with any random subsample.

The analysed subsample of point counts detected 40.8% of all species, compared with a mean (±sd) of 41.4% (±1.8) for all random subsamples. The analysed subsample is therefore within half a standard deviation of the mean for all random subsamples, whereas point counts in BFR detected 59.8% of species – more than any of the random subsamples generated from the CNP data. This shows that our conclusions about the variability of effectiveness of this method between the two locations are not sensitive to the process of random subsampling.

Furthermore, without any correction for unequal sampling effort, mist-netting detected 33.2% of species in CNP, and point counts detected 48.7%. Therefore, even if all data are included the differences in each method’s efficacy are apparent.

In summary, randomly subsampling these survey data equalised survey effort between locations, without affecting indicators of each method’s efficacy, as the difference between CNP and BFR was so much greater than the variation present in random subsamples within CNP.
